# Performance Analysis of the Ironless Inductive Position Sensor in the Large Hadron Collider Collimators Environment

**DOI:** 10.3390/s151128592

**Published:** 2015-11-12

**Authors:** Alessandro Danisi, Alessandro Masi, Roberto Losito

**Affiliations:** CERN—European Organization for Nuclear Research, Route de Meyrin, Geneva CH-1211, Switzerland; E-Mails: alessandro.masi@cern.ch (A.M.); roberto.losito@cern.ch (R.L.)

**Keywords:** ironless inductive position sensor, harsh environment, linear variable differential transformer, LHC, collimators, linear position sensing

## Abstract

The Ironless Inductive Position Sensor (I2PS) has been introduced as a valid alternative to Linear Variable Differential Transformers (LVDTs) when external magnetic fields are present. Potential applications of this linear position sensor can be found in critical systems such as nuclear plants, tokamaks, satellites and particle accelerators. This paper analyzes the performance of the I2PS in the harsh environment of the collimators of the Large Hadron Collider (LHC), where position uncertainties of less than 20 µm are demanded in the presence of nuclear radiation and external magnetic fields. The I2PS has been targeted for installation for LHC Run 2, in order to solve the magnetic interference problem which standard LVDTs are experiencing. The paper describes in detail the chain of systems which belong to the new I2PS measurement task, their impact on the sensor performance and their possible further optimization. The I2PS performance is analyzed evaluating the position uncertainty (on 30 s), the magnetic immunity and the long-term stability (on 7 days). These three indicators are assessed from data acquired during the LHC operation in 2015 and compared with those of LVDTs.

## 1. Introduction

The Large Hadron Collider (LHC) is the largest synchrotron ever built [1]. Its combined beam power is potentially capable of melting 1 ton of copper. To protect the superconducting magnets from abnormal beam losses, electromechanical devices (the LHC collimators) are used to reduce the excess losses and to clean the beam halo [2]. 

**Figure 1:**
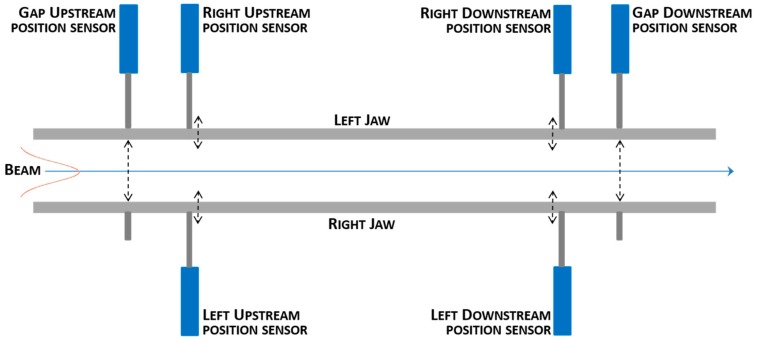
Diagram of the six linear position sensors installed in the LHC collimators.

To perform this delicate task, the LHC collimators make use of a pair of 1-meter-long jaws (which can be made of different materials according to the specified collimator, namely, graphite, tungsten, carbon composites [2]). The jaws are aligned with the beam and can be moved in the transversal plane (Figure 1), so as to make contact and absorb the halo particles [2,3]. An accurate motorization system (involving stepper motors and related custom drives [4,5]) is used to perform the positioning of the jaws at the correct point. In addition, six Linear Variable Differential Transformers (LVDTs) are used as linear position sensors (Figure 1), in order to have a closed-loop control and assure very high-precision measurements (*i.e.*, <20 µm on a 60 mm position range) [4].

These linear position sensors are meant to work in a particularly harsh environment. As a matter of fact, the high-precision specification on the linear position sensors is to be achieved in presence of nuclear radiation (coming from the interaction of the beam with the jaws’ material) and stray magnetic fields (coming from high-current cables and motors placed nearby) [6]. In addition, the sensors should guarantee a long lifetime (*i.e.*, several years) and robustness (a secondary specification which comes from the radiation environment). This set of specifications are similar to the ones which can be found in nuclear plants or fusion experiments [7].

For the particularly demanding harsh environment specifications under consideration, the Linear Variable Differential Transformer (LVDT) has been chosen for the real-time monitoring of the LHC collimator jaws’ position [4,8]. This sensor can be designed to be radiation-hard with suitable insulation techniques [9], provides outstanding precision (less than 1 µm with suitable conditioning electronics [8]) and is a robust long-lifetime solution [10,11]. 

However, despite its outstanding performance in terms of precision, the LVDT exhibited a particular sensitivity to external magnetic fields, which became evident during the sensor operation on the collimators. As a matter of fact, the magnetic field generated by high-current cables placed less than 2 m away from come collimators have shown to affect the LVDT position reading. A position drift of several hundreds of micrometers is reported, reproducible also with laboratory equipment [6,12,13,14].

Since such big drifts are not compatible with the stringent LHC collimators requirements mentioned above, the Ironless Inductive Position Sensor (I2PS) has been proposed as a valid alternative to LVDTs when external magnetic fields are present and immunity is required. The electromagnetic model of the sensor, with its low-frequency (e.g., magnetic coupling) and high-frequency (e.g., proximity effect) implications, has been described in detail in [15,16]. The real-time algorithm chosen to read the sensor has been introduced in [17], whereas the thermal effects have been analyzed in detail in [18]. Finally, an optimization procedure of the sensor for the LHC collimators application has been presented in [19], where the I2PS general design guidelines have been drawn. In this paper, the final performance of the I2PS sensor in the LHC harsh environment during the accelerator operation are analyzed. The necessary modifications to the existing LHC collimator low level control system are addressed and the I2PS magnetic immunity during LHC operation is finally shown.

## 2. The Ironless Inductive Position Sensor (I2PS)

**Figure 2:**
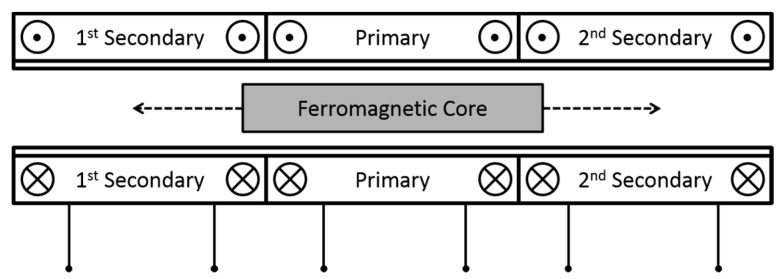
Working principle of a Linear Variable Differential Transformer (LVDT).

The LVDT sensor is characterized by a contactless working principle (which leads to a long lifetime), where a primary coil is excited and two secondary-coil voltages are read (Figure 2). A ferromagnetic permeable core is mechanically coupled with the movable jaw and its movement inside the sensor varies the secondary amplitudes [20]. A differential or ratiometric [21] reading of the voltages brings to the extraction of the position. 

In presence of external DC or slowly-varying magnetic fields, the LVDT reading has been shown to drift from its correct value, following the time evolution of an external magnetic field generated by a high-current cable placed in proximity (<2 m) of the LHC collimators [19,22]. This magnetic drift can be of the order of some hundreds of micrometers, a value which of course is in contrast with the abovementioned high-precision specification of the LHC collimators. Detailed studies from analytical, numerical and experimental viewpoints [13,14,23] highlighted that this phenomenon is intrinsically dependent from the non-linearity of the B-H curve [24] of the ferromagnetic materials of the LVDT sensor (namely, the core and the external housing, typically made of Ni-Fe alloy and steel respectively [25,26]). This phenomenon cannot be avoided, but only reduced with techniques as magnetic shielding or primary voltage polarization [12]. However, these countermeasures may not be always feasible for space and design constraints and only partially reduce the effects [12,19]. 

**Figure 3:**
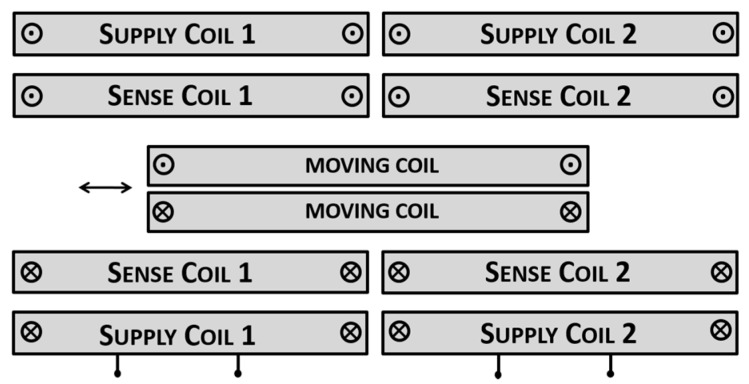
Working principle of an Ironless Inductive Position Sensor.

The working principle of the I2PS is based on the spatial modulation of the magnetic coupling between cylindrical coaxial coils (as for standard LVDTs), with the variation being induced by a moving coil (instead of a ferromagnetic part), which is short-circuited and free to move longitudinally (Figure 3). Two supply coils, fed with two equal and opposite sinusoidal currents, provide the excitation field, whereas the sense coils voltages are read with a differential (e.g., ratiometric) technique, as for common LVDTs. Being the moving coil short-circuited, the related induced current generates a counter-acting magnetic fields which decreases the voltage on the sense coil facing it, whereas increases the other sense voltage. The voltage difference is a function of the moving coil position and nullifies only when the moving coil is in the center, since in this case its induced current is null.

Analyzing the I2PS working principle, one can easily discover many points in common with standard LVDTs. This includes the contactless sensing, the virtually infinite resolution, the similarity of the potential reading technique, and the use of cylindrical coils. These features indicate the required properties of a long-lasting and robust device. A more quantitative comparison between these 2 linear position sensors is given in Table 1. In addition, radiation-hardness can be achieved using insulation techniques [27] and high-precision readings can be obtained by adopting the same reading technique (*i.e.*, ratiometric) as for standard LVDTs. Finally, since the structure is free from ferromagnetic materials, the effective B-H curve is never non-linear. Therefore, the intrinsic immunity to external fields is a key property. 

**Table 1:** Main features of LVDT and I2PS.

Feature	LVDT	I2PS
Working Principle	Inductive	Inductive
Supply amplitude range	2–5 V	10–50 mA
Sensing voltage amplitude	~3–5 V	~7–10 V
Frequency range	1–5 kHz	1–5 kHz
Stroke range	±30 mm	±30 mm
Dimensions	Diameter: 21 mm, Length: 210 mm	Diameter: 27 mm, Length: 230 mm
Materials	Ni-Fe alloy, copper (coils), magnetic steel.	Plastics, copper (coils), non-magnetic steel.

## 3. The I2PS Network

### 3.1. Control System Architecture 

The I2PS has been successfully tested with detailed laboratory experiments [22], which have been followed by prototyping and a first series production. Following the positive results of such studies, the first installation of I2PS sensors in the LHC collimators has taken place in November 2014 and involved the replacement of 10 LVDTs on five different collimators along the LHC ring, in order to finally benchmark the sensor effectiveness and its harsh-environment specifications. The five collimators have been selected because they were strongly affected by the LVDT interference. Two of the six LVDTs have been replaced with I2PS sensors in each collimator, leaving the remaining four to compare the performance. 

The LHC collimator low level control system is made of two crucial units: the Motor Drive Control (MDC) and the Position Readout and Survey system (PRS) [4]. Each unit is implemented with custom software deployed on a real-time unit, which involves the use of acquisition, motion and synchronization boards. The MDC performs the tasks related to the movement of the collimator jaws, whereas the PRS mainly monitors the linear position sensors and generates the beam interlocks when the position reading is out of specified limits [2].

Each MDC-PRS couple can implement a control system for three different collimators [4]. The control system hardware also entails custom generation/acquisition boards for the conditioning of the sensors’ voltages and stepper motor drivers for long-cable motor control. This hardware is commonly located in specified caverns several hundred meters away from the collimator itself [4].

### 3.2. I2PS Integration

**Figure 4:**
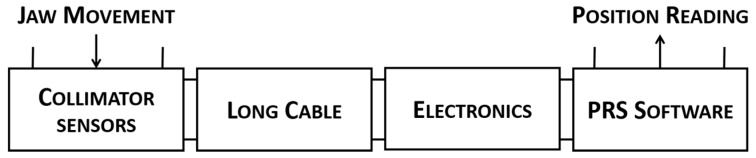
Illustration of the stages which are needed to perform the position measurement of the LHC collimator jaws.

The presence of an I2PS in a collimator mainly affects the PRS-side of the control system. With reference to Figure 4, some interventions have been necessary to integrate the I2PS position reading into the existing LHC collimators low level control system.

**Figure 5:**
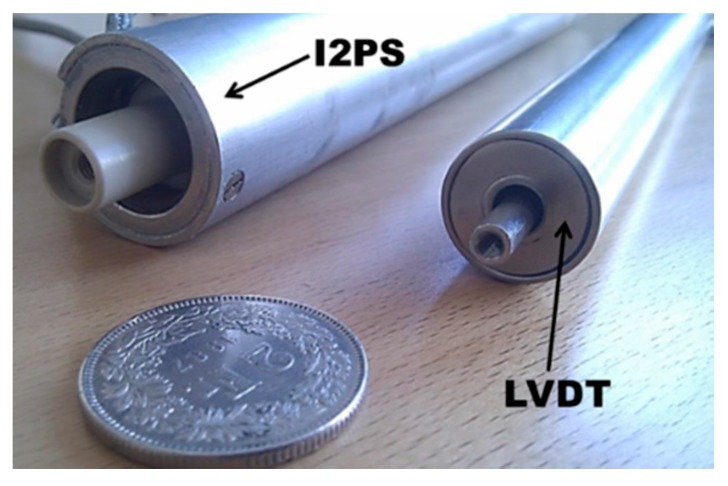
Visual comparison of an Ironless Inductive Position Sensor (I2PS, left) with a Linear Variable Differential Transformer (LVDT, right).

On the collimator itself, only slight mechanical changes have been performed. As a matter of fact, the I2PS sensor is quite similar to LVDT in terms of shape (Figure 5); it only exhibits slightly larger diameter (27 mm against 21 mm) and length (23 cm against 21 cm) [19,22]. Therefore the mechanism for the clamping of the sensor and the alignment of moving parts have been just adapted to such variations.

Since standard LVDTs have 8-pin outputs (two for primary excitation, four for secondary readings and two additional ones for a built-in PT100 temperature sensor), no modifications have been necessary to the cable connecting the signals to the conditioning electronics. As a matter of fact, the I2PS sensor has been designed to deliver an 8-pin output as well (two for the supply coils connected in series, four for the sense coils and two additional ones for the temperature compensation [25]). It has to be stated that such cables can be up to 1 km long in certain LHC ring locations [8], therefore introducing additional noise and losses.

The conditioning electronics had to be designed differently with respect to LVDTs. As a matter of fact, the I2PS is fed with a current signal, whereas the LVDT is commonly excited with a voltage sinewave [17]. For this reason, and to provide a stable current with different loads, a custom current excitation board has replaced the usual LVDT generation board in the control system rack. It has to be stated that the I2PS voltages are acquired with an attenuation factor of 0.5, since the PRS acquisition boards are 5-V full scale, whereas the I2PS voltages are commonly in the 10-V scale. Since the uncertainty of the ratiometric operation depends on the voltage amplitudes (the ratiometric one is nonlinear operation) such attenuation may cause a slight deterioration of the overall sensor uncertainty [19,22]. However, this attenuation has been introduced in order to further decrease the impact of the I2PS installation on the PRS hardware and on the control system in general. In principle, it can also be removed and *ad-hoc* acquisition boards can be installed in the dedicated controllers associated to the selected collimators where I2PS sensors are targeted to be installed. Of course, this change would have a higher impact on the hardware, and for this first I2PS installation it has been skipped, whereas it will be taken under consideration for possible future upgrades.

**Figure 6:**
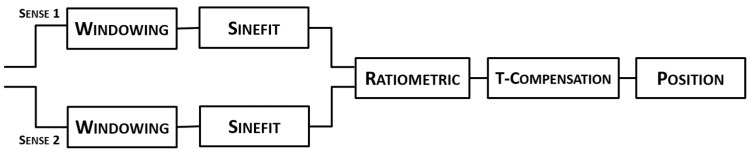
Scheme of the I2PS reading technique. With respect to standard LVDTs, the additional blocks used for the I2PS are the windowing and the temperature compensation.

The modification demanded to the PRS software are instead connected to the sensor’s reading: implementation of the temperature compensation technique [18], signal conditioning prior to the Sinefit demodulation and ratiometric reading [17]. The steps for the reading are depicted in Figure 6. The sampling rate of the signals, as well as the buffer duration and demodulation remain unaltered from that of the standard LVDT conditioning [8]. These operations are performed in the dedicated real-time window which defines the survey rate of the control system [4]. 

With these minor changes to the LHC collimator control system, the integration of the I2PS sensor is complete. As there are many similarities between the I2PS and the LVDT (shape, dimensions, type of signal, frequency, reading technique), the modifications cause minimal impact from hardware and software viewpoints. The choice of a sensor based on a different working principle and/or with different shape or dimensions (e.g., interferometric sensor) would have entailed a much higher impact on the hardware and software integration. From this point of view, the I2PS was the best solution to add the magnetic immunity to the LHC collimator position sensing system. 

## 4. Analysis of Sensor Performance

The performance of the Ironless Inductive Position Sensor has been analyzed by taking into account three main variables: the position uncertainty, the magnetic immunity and the long-term stability. The resistance to radiation has been instead tested with a dedicated procedure in an irradiation facility before the installation [28] and therefore will not be addressed in this work. 

It has to be stated that the analysis here presented has been performed on data taken during operation of the LHC after the Long Shutdown 1 (LS1) in April 2015. Therefore, all systems are operating at their nominal conditions and this analysis provides a clear indication on the I2PS network behavior in the LHC harsh environment. This estimation is certainly different and much more relevant to the application with respect to a performance analysis carried out with laboratory experiments.

### 4.1. Position Uncertainty

Such uncertainty has been measured by taking into account different time windows (corresponding to different collimator positions, which in turn correspond to different beam modes [2]) and computing the standard deviation of the position measured by the sensor on a target collimator in a period of 30 s with a survey rate of 100 Hz (1 position measurement each 10 ms), yielding to 3000 position samples. Assuming normal distribution, the corresponding uncertainty has been taken as twice the computed standard deviation, according to the definition of type-B uncertainty with coverage factor 2. The results have been compared with the LVDT values on the same collimator and are shown in Table 2. 

It is noticeable that the I2PS standard deviation is below 1 µm, therefore the related uncertainty is well within the specifications of the LHC collimator low level control system. These values are only slightly higher than the corresponding LVDT results, confirming the adequate uncertainty performance of the I2PS. As anticipated in Section 3, the reason for this slight. Difference is due to the voltage attenuation which is implemented in the acquisition electronics.

**Table 2:** Standard deviation of the measured position over 3000 samples.

Sensor	Type	Standard Deviation/µm	Position Uncertainty/µm
1	LVDT	0.3	±0.6
2	LVDT	0.5	±1.0
3	I2PS	0.7	±1.4
4	LVDT	0.6	±1.2
5	I2PS	0.9	±1.8
6	LVDT	0.4	±0.8

### 4.2. Magnetic Immunity

The unique feature of magnetic immunity of the I2PS has been verified by monitoring the position reading during a current cycle of the LHC magnets. The high current flowing in the cables located less than 2 m away from the collimator generates the external magnetic field. The LVDT and I2PS position readings have been compared during these time windows, while the collimator jaws were not moved and therefore the position reading should have returned a constant value. 

**Figure 7:**
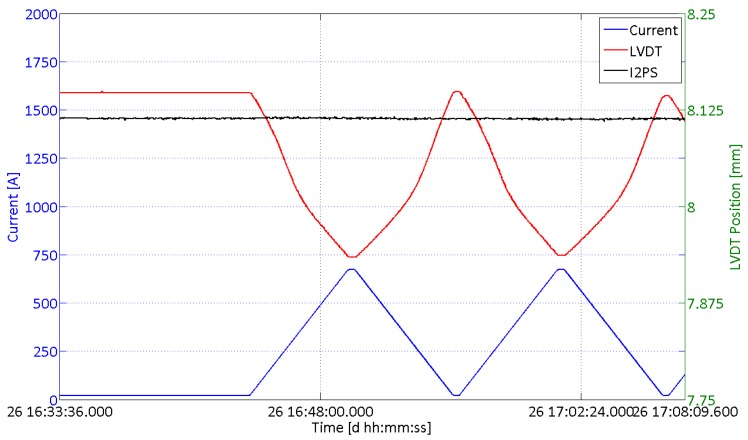
I2PS and LVDT position readings during a magnet current cycle, generating an external field in proximity of the linear position sensors. The time period is 26 May 2015, during a span of 35 min of LHC operation.

The results are shown in Figure 7. It is noticeable that the time evolution of the LVDT reading is perfectly synchronized with the external current signal (*i.e.*, with the external field) and the position drifts from its initial value. The magnitude of this particular drift overcomes 200 µm, giving an illustrative example of the criticality of this issue for the LHC collimators. The I2PS reading, instead, remains at its initial value, reporting no influence coming from the external field and returning a constant value, as it is expected when the jaws are not in movement. The other I2PS sensor installed on the same collimator gave the same results, whereas the other LVDTs on the same collimators reported very similar drifts. This result confirms the effectiveness of the I2PS sensor in terms of magnetic immunity when compared to standard LVDTs on the same collimator. The same results are also observed on the other collimators equipped with the I2PS, in different positions, whenever a magnetic interference on standard LVDTs is reported to happen. 

### 4.3. Long-term Stability 

This value has been defined as the standard deviation of the measured position in a wide time span (7 days, with a survey rate of 100 Hz, leading to a totality of 6 × 10^7^ samples). The long monitoring time allows accounting for all long-term issues, e.g., ambient temperature variations, electronics drifts, collimator local temperature and supply current stability. If the collimator jaws move during this span, the monitoring stops and resumes only when the jaws are back to their previous position. Therefore, a long-term stability defined in this way includes also an indication of the position repeatability, revealing as a useful statistical tool to assess the long-term performance of the I2PS. 

The results are presented in Table 3, together with values coming from LVDTs installed on the same collimator. The I2PS long-term stability has shown to be slightly worse with respect to LVDTs, but within the LHC collimators requirements in three cases out of five. The length of the cable is also listed, since an evident correlation can be drawn between the latter and the average deviation: the longer the length, the higher the deviation. However, as anticipated, at least on three out of five collimators, the standard deviation on the long term is well below the 20-µm uncertainty requirements of the LHC collimators, even if one chooses to adopt a coverage factor of 2. The reasons for higher long-term deviations may be due to thermal variations which are not compensated for, or to a more probable non-constant supply current amplitude due to load variation and/or thermal effects on the long cable. This hypothesis shows more consistence with the observed correlation with the cable length. The slightly higher I2PS deviation (with respect to LVDT counterpart) in the first three collimators is due to the voltage attenuation worsening the ratiometric uncertainty. Similar results have been obtained for different jaw positions.

**Table 3:** Long-term stability of I2PS and LVDT sensors on 5 collimators.

Collimator	Cable Length (m)	Long-term Standard Deviation (µm)	
		LVDT	I2PS
1	180	2	3
2	293	5	7
3	380	7	9.5
4	452	3	20
5	569	10	25

## 5. Conclusions and Outlook

The Ironless Inductive Position Sensor has been installed on five different collimators along the LHC tunnel to benchmark its properties of magnetic immunity and high-precision, in replacement of 10 selected LVDTs which have been found to be particularly sensitive to external magnetic fields. 

The performance of the I2PS have been analyzed in this paper from the viewpoints of uncertainty, magnetic immunity and long-term stability. The observed uncertainty is similar to that of standard LVDTs, whereas the magnetic immunity has been fully benchmarked with observations during the LHC run 2, where the I2PS showed to be insensitive to an external field which instead was giving a 200-µm drift on a standard LVDT on the same collimator. The long-term stability study gave very good results on three out of five of the selected collimators, whereas on the other two additional studies on the generation electronics have to be addressed in order to further improve the stability of the supply current. 

Given the very good results of the I2PS performance analysis presented in this paper, a future campaign on increasing the number of I2PS in the LHC is foreseen, as well as a deeper study of the conditioning electronics with long cables. The uncertainty can be further improved by removing the attenuation at the acquisition side. In this case, 10-V full-scale boards have to be chosen to replace the current 5-V ones dedicated to LVDTs. 
